# Clinical Attitudes, Behaviors and Perceived Stress Towards the COVID-19 Pandemic: A Questionnaire Survey among Swiss Dentists

**DOI:** 10.3290/j.ohpd.b3500619

**Published:** 2022-10-19

**Authors:** Panagiotis Gardelis, Rebecca Ebersberger, Alkisti Zekeridou, Catherine Giannopoulou

**Affiliations:** a Research Assistant, Division of Regenerative Dental Medicine and Periodontology, University Clinics of Dental Medicine, Faculty of Medicine, University of Geneva, Geneva, Switzerland. Substantially contributed to analysis and interpretation of the data, drafted the paper, critically revised the paper, approved the final version.; b Master’s Student, University Clinics of Dental Medicine, Faculty of Medicine, University of Geneva, Geneva, Switzerland. Substantially contributed to study design, acquisition, analysis and interpretation of the data, drafted the paper, approved the final version.; c Research and Teaching Fellow, Division of Regenerative Dental Medicine and Periodontology, University Clinics of Dental Medicine, Faculty of Medicine, University of Geneva, Geneva, Switzerland. Substantially contributed to analysis and interpretation of the data, drafted and critically revised the paper, approved the final version.; d Associate Professor, Division of Regenerative Dental Medicine and Periodontology, University Clinics of Dental Medicine, Faculty of Medicine, University of Geneva, Geneva, Switzerland. Substantially contributed to study design, analysis and interpretation of the data, drafted the paper, approved the final version.

**Keywords:** dentistry, COVID-19, questionnaire, awareness, protective measures, stress

## Abstract

**Purpose:**

To investigate dentists’ work conditions, awareness, protective measures, economic effects and perceived stress during the first two waves of the COVID-19 pandemic.

**Materials and Methods:**

This cross-sectional survey was conducted among 126 dentists working in the French-speaking part of Switzerland, in particular in the Cantons of Vaud and Geneva. Data consisted of the answers to 40 questions assessing the knowledge, attitudes, workload and mental condition of the dentists during the first 2 waves of the COVID-19 pandemic.

**Results:**

Swiss dentists received sufficient information about the COVID-19 pandemic and implemented protective measures. Differences were found between the 1st and the 2nd wave concerning the workload; during the first wave, the workload was low for the majority of dentists (60%), whereas during the second, it was moderate (53.4%) or high (41.3%). During both waves, the mental burden was also important, and was related mainly to financial issues and fear of infection.

**Conclusions:**

This survey reported that Swiss dentists were, in general, satisfied with the transmission of precise operating guidelines during the pandemic. However, a considerable psychological impact, mainly during the first wave, was revealed. With the implementation of proper strategic measures during the COVID-19 outbreak, dental practitioners will be prepared for future global health-care disruptions.

On December 2019, the World Health Organization (WHO) reported that a new virus had emerged in the Republic of China, causing pneumonia.^25^ The disease called COVID-19 is caused by the coronavirus SARS-CoV-2 (Severe Acute Respiratory Syndrome Corona Virus Type 2). The initial outbreak spread rapidly and several other countries were soon affected. In Switzerland, the first case was recorded in Ticino in February 2020.^15^ On 11 March, the WHO declared a pandemic situation and countries were called to take measures such as screening, isolation and tracing of the population to prevent infection.^24^ All sectors of public life were affected, with an unprecedented lockdown and restricted access to public spaces, services and all manner of establishments.

In Switzerland, in view of the rapid spread, the Federal Council decided on 16 March 2020 to close down all shops, restaurants, bars and other entertainment and leisure establishments, introduce border controls for the neighbouring countries and request the help of the army to support the Cantons until 19 April 2020.^7^

It quickly became evident that the virus is highly transmissible through virus-loaded droplets and aerosols. These are generated not only while speaking, coughing or simply breathing, but also during dental treatment; thus dentists and their team were soon considered at higher risk than the general population. Dental practice was restricted or even stopped. The Swiss Society of Odontostomatology (SSO) published a document on “Guidelines for operating in a dental practice during the COVID-19 pandemic” which included all information on how to protect the health of vulnerable patients and health care professionals by implementing appropriate protective measures.^2^ As expected, the reduction or cessation of work in some practices led to financial and health concerns. This is why, on 14 April, the SSO initiated the “Smart Restart” program to allow the Swiss population to access dental care again.^18^

After almost 2 years from the start of the pandemic, five waves of COVID-19 infection have emerged with new variants of the virus causing disease, such as the Delta mutation and the Omicron mutation from South Africa. In addition, a vaccine became availble at the beginning of 2021, and currently the 3rd vaccination is available to the entire population as a booster. In parallel, as oral health and in particular periodontitis have been shown to significantly impact several systemic diseases/conditions, a new area of research was developed on the role of periodontal disease in the severity of COVID-19 infection.^11,12,19^

The present survey was conducted in spring 2021. The aim was to investigate the impact of the COVID-19 pandemic on dentists working in the French-speaking part of Switzerland. As this year has been marked by other global events leading to concerns about security, combined with strict measures during the first two waves of the pandemic, our questionnaire focused on dentists’ work conditions, awareness, protective measures, economic effects and perceived stress during this period.

## Materials and Methods

### Sample and Data Collection

The present cross-sectional survey was conducted in spring 2021. A total of 314 dentists practicing in the French-speaking part of Switzerland (the Cantons of Geneva and Vaud) were randomly selected to participate. An electronic version of the questionnaire together with an information sheet was e-mailed to registered dentists working in private or public clinics. Both general and specialist dentists were enrolled. The questionnaire was e-mailed again three months later in order to obtain a sufficient number of answers.

### Questionnaire

The questionnaire consisted of 40 items, some of them adapted from existing questionnaires used in similar studies.

The questionnaire was divided into 4 sections.^4,5,14^ The first part concerned personal data (age, sex, practice location [Canton], years since graduation, university which granted the degree, the obtained specialty [general dentistry, orthodontics, periodontics, prosthodontics or maxillofacial surgery]) and number of working hours/week. The second part consisted of 8 items regarding the awareness and attitudes adopted by the professionals during the pandemic. The third part consisted of 14 items assessing the workload during the two waves compared to the situation before the outbreak of the virus, as well as the economic impact. The final part consisted of 8 items to assess the psychological effect on the dental professionals. For the workload and mental burden, subjective self-reported answers were given by the participants as “high”, “medium” or “low”.

A preliminary questionnaire was constructed and pre-tested on a small group of dentists (N = 6).

### Statistical Analysis

We used descriptive statistics with frequency and percentages to evaluate the dentists’ answers.

## Results

### Demographics

A total of 314 questionnaires were sent to dentists in spring 2021 and 126 were completed (40% response rate). As shown in Table 1, the participation of the dentists was similar for both Cantons (51.6% in the Canton of Vaud and 48.4% in the Canton of Geneva). Responses were received from 66.7% (N = 84) male and 33.3% (N = 42) female dentists. The mean age was 51 years (range 26-79), the years of work experience varied from 1 to 50, and the majority reported not having any postgraduate qualification (73.8%). 82.2% of the dentists had graduated from a Swiss university and 17% from universities of other European countries. Most of the dentists (81.7%) reported working 38 hours/week or more.

**Table 1:** Personal characteristics of responders (N = 126)

Personal characteristics	
**Gender**	
Female Male	42 (33.3%) 84 (66.7%)
**Age in years**	
Range 26-50 Range 51–75 >75	55 (43.7%) 70 (55.5) 1 (0.8%)
**Marital status**	
Married Single Widowed No answer	71 (56.3%) 50 (39.7%) 1 (0.8%) 4 (3.2%)
**Graduation from university in**	
Switzerland Other country No answer	104 (82.2%) 22 (17%) 1 (0.8%)
**Years of experience**	
1–10 years 11–20 years 21–30 years 31–40 years >40 years No answer	20 (15.9%) 26 (20.6%) 28 (22.2%) 39 (31%) 11 (8.8%) 2 (1.5%)
**Years of practice in Switzerland**	
1–10 11–20 21–30 31–40 >40 No answer	24 (19%) 21 (16.7%) 33 (26.2%) 35 (27.8%) 12 (9.5%) 1 (0.8%)
**Specialty (multiple answers possible)**	
General Dentistry Periodontology Orthodontics Reconstructive Dentistry Implantology Pediatric Dentistry Others No response	93 (73.8%) 11 (8.7%) 11 (8.7%) 15 (11%) 4 (3.2%) 3 (2.4%) 3 (2.4%) 1 (0.8%)
**Hours worked per week**	
4–10 11–20 21–38 >38 No answer	2 (1.6%) 3 (2.4%) 17 (13.5%) 103 (81.7%) 1 (0.8%)
**Practice location (Canton)**	
Vaud Genève	65 (51.6%) 61 (48.4%)
**Region of practice**	
Urban Rural No answer	113 (89.7%) 12 (9.5%) 1 (0.8%)

Expressed in number of answers (percentages).

Data on the dentists’ awareness and practices during the COVID-19 pandemic are shown in Table 2. Information on the management of patients was received mainly through professional associations (81.7%), followed by various websites (48.4%) and colleagues (23%). Only 7.9% of the participants reported attending online information sessions (videoconferences). The majority of the dentists (86.5%) felt sufficiently informed about COVID-19. However, opinions on the relative risk of infection in the dental practice vs a supermarket were divided equally between “higher” or “lower”. Furthermore, 88.1% of the responders considered themselves at risk for COVID-19 infection. Half of the responding dentists estimated that the management of patients was better during the 2nd wave, mainly because of the availability of the required protective material and more precise guidelines. As shown in Table 2, the majority of the participants in our survey implemented regular disinfection of the surfaces, ventilation of the working area and limitation of the number of accompanying persons. In terms of PPE, FFP2/KN95 masks and protective glasses were worn by 86.5% and 82.5% of the participants, respectively. A lower percentage of dentists used a protective face shield (68.3%), or wore a bonnet (47.6%) or a gown (34.1%) However, only half of the participants reported being satisfied with these measures, while the other half reported being “more or less” satisfied.

**Table 2:** Awareness and practices of the dental professionals during the COVID-19 pandemic (N = 126)

Knowledge/attitude	
**Do you consider yourself as a person at risk of COVID-19?**	
Yes No	111 (88.1%) 15 (11.9%)
**What was your source of information about COVID-19?**	
Professional associations Websites Colleagues Videoconferences Television Others (Cantonal dentist, University…)	103 (81.7%) 61 (48.4%) 29 (23%) 10 (7.9%) 6 (4.8%) 11 (6.4%)
**Was the information about COVID-19 clear enough for your daily paractice?**	
Yes No No answer	109 (86.5%) 15 (11.9%) 2 (1.6%)
**Do you think that the risk of contamination in a dental clinic is**	
Higher than that in a supermarket? Lower than that in a supermarket? Similar to that in a supermarket? No answer	55 (43.6%) 52 (41.3%) 18 (14.3%) 1 (0.8%)
**Did you notice any differences in the 2nd wave compared to the 1st wave concerning the management of patients?**	
Yes No No response	63 (50%) 60 (47.6%) 3 (2.4%)
**If yes, which ones? (multiple answers possible)**	
Materials were available Guidelines were clearer Better care More confident patients Less anxiety in patients No response	55 (43.6%) 50 (40%) 39 (31%) 3 (2.4%) 1 (0.8%) 56 (44.4%)
**Precautions you have applied and continue to apply in your practice (multiple answers possible)**	
Regular disinfection of surfaces Ventilation of the environment Limitation of accompanying persons Wearing of FFP2/KN95 masks Wearing of protective glasses Limiting the flow of patients Telephone or reception questionnaire Protective face shield Patients rinsing with antiseptic solution Working with four hands Wearing surgical masks Wearing of bonnet Reducing aerosol production Taking patients’ temperatures Wearing a gown Use of anti-retraction handpieces Sterile gloves UV-C air purifiers Rubber-dam at the start of treatment	26 (100%) 120 (95.2%) 117 (92.9%) 109 (86.5%) 104 (82.5%) 94 (74.6%) 91 (72.2%) 86 (68.3%) 85 (67.5%) 79 (62.7%) 67 (53.2%) 60 (47.6%) 59 (46.8%) 48 (38.1%) 43 (34.1%) 19 (15.1%) 17 (13.5%) 1 (0.8%) 1 (0.8%)
**Are you satisfied with the measures in place for the care of patients during the pandemic?**	
Yes No More or less No answer	66 (52.4%) 6 (4.8%) 52 (41.3%) 2 (1.5%)

Expressed in number of answers (percentages).

During the lockdown, 77.8% of the dentists continued to provide emergency care either as a result of a personal decision or because of the State’s request (Table 3). The workload before the COVID-19 epidemic was reported as “high” by 2/3 of the participants and “medium” by almost 1/3. During the 1st wave, the workload was “low” for 2/3 of the dentists and became “high” again during the 2nd wave for 41.3% of the dentists. At the same time, almost half of the dentists reported an income loss up to 25% compared to the pre-COVID situation. Although the State provided financial support for professionals, this was described as sufficient by only 1/3 of the responders.

**Table 3:** Workload and economic impact of the COVID-19 pandemic related to the 2 waves (N = 126)

Impact on your practice	
**How many patients (on average) did you see per week before the COVID-19 pandemic started?**	
1–50 51–100 >100 No answer	76 (60.3%) 27 (21.4%) 9 (7.2%) 14 (11.1%)
**When the lockdown was imposed, did you continue to provide emergency care?**	
Yes No No answer	98 (77.8%) 27 (21.4%) 1 (0.8%)
**If so, was it as a result of:**	
A personal decision? A request from the state? No answer	56 (44.4%) 47 (37.3%) 23 (18.3%)
**In the 2nd wave, compared to the period before COVID-19, did the patient flow:**	
Remain stable? Decrease? Increase? No answer	55 (43.6%) 52 (41.3%) 17 (13.5%) 2 (1.6%)
**Workload before the 1st wave**	
High Medium Low No response	82 (65.1%) 37 (29.4%) 6 (4.7%) 1 (0.8%)
**Workload during the 1st wave**	
High Medium Low No response	21 (16.7%) 28 (22.2%) 76 (60.3%) 1 (0.8%)
**Workload during the 2nd wave**	
High Medium Low No response	52 (41.3%) 68 (53.9%) 4 (3.2%) 2 (1.6%)
**Have you been infected with COVID**–**19?**	
Yes No No answer	19 (15.1%) 105 (83.3%) 2 (1.6%)
**Have members of your team been infected with COVID?**	
Yes No No answer	67 (53.2%) 56 (44.4%) 3 (2.4%)
**If yes, how many persons were infected in relation to the total number of people working in your clinic?**	
0%–24% 25%–49% 50%–74% 75%–100% No answer	34 (27%) 18 (14.3%) 6 (4.8%) 2 (1.5%) 66 (52.4%)
**Were members of your team under lockdown?**	
Yes No No answer	78 (61.9%) 46 (36.5%) 2 (1.6%)
**If yes, how many persons were under lockdown in relation to the total number of people working in your clinic?**	
0%–24% 25%–49% 50%–74% 75%–100% No answer	26 (20.6%) 23 (18.3%) 5 (4%) 10 (7.9%) 62 (49.2%)
**Was the financial support from the state sufficient?**	
No Yes Moderately No answer	44 (35%) 42 (33.3%) 29 (23%) 11 (8.7%)
**In percent, how much has your income decreased between the years 2019 and 2020 (calculating 2019 as 100%)?**	
No change By 1% to 25% By 26% to 50% By > 50% No answer	15 (11.9%) 68 (54%) 19 (15.1%) 1 (0.8%) 23 (18.2%)

Expressed in number of answers (percentages).

As shown in Table 4, before the COVID-19 outbreak, the mental burden (in terms of stress and anxiety) was “high” for approximately 1/3 of the dentists. During the 1st wave, more than half of the responders had a high mental burden, mainly for financial reasons (60.3%), followed by fear of infection (47.6%), lack of knowledge (33.3%) and lack of guidelines (27%). During the 2nd wave, the psychological load was “medium” for half of the dentists, and only 24.6% continued to report high psychological load. Financial reasons were reported as the major source of stress during the 2nd wave by 41.3% of the dentists, followed by fear of infection (40.5%) and family reasons (23%). Lack of knowledge and lack of guidelines were no longer major sources of the perceived stress by the dentists. The majority of the dentists were not particularly stressed and felt confident when providing dental treatment to their patients.

**Table 4:** Sources of stress during the COVID-19 pandemic (N = 126)

Source of stress	
**Mental load before the 1st wave**	
High Medium Low No response	46 (36.5%) 58 (46%) 20 (15.9%) 2 (1.6%)
**Mental load in the 1st wave**	
High Medium Low No response	69 (54.8%) 35 (27.7%) 19 (15.1%) 3 (2.4%)
**Mental load in the 2nd wave**	
High Medium Low No response	31 (24.6%) 61 (48.4%) 30 (23.8%) 4 (3.2%)
**Major sources of stress before the 1st wave (multiple responses possible)**	
Finances Competition Family Amount of work Employee and facility management Number of patients Health Quality of care State paperwork Psychological stress No response	46 (36.5%) 31 (24.6%) 24 (19.1%) 12 (9.52%) 3 (2.4%) 2 (1.6%) 3 (2.4%) 4 (3.2%) 1 (0.8%) 1 (0.8%) 25 (20%)
**Major sources of stress during the 1st wave (multiple responses possible)**	
Finances Infection Lack of knowledge Lack of guidelines Family Competition Uncertainty of pandemic duration Others No reponse	76 (60.3%) 61 (47.6%) 42 (33.3%) 34 (27%) 27 (21.4%) 6 (4.8%) 4 (3.2%) 16 (12.8%) 25 (19.8%)
**Major sources of stress during the 2nd wave (multiple responses possible)**	
Finances Infection Lack of knowledge Lack of guidelines Family Competition Others No response	52 (41.3%) 51 (40.5%) 10 (8%) 4 (3.2%) 29 (23%) 15 (11.9%) 15 (11.9%) 10 (7.9%)
**Do you feel stressed when treating a patient? (1 = not stressed and 5 = very stressed)**	
1 2 3 4 5 No answer	78 (61.9%) 28 (22.2%) 16 (12.7%) 3 (2.4%) 0 (0) 1 (0.8%)
**Do you feel confident when treating your patients? (1 = not at all confident and 5 = very confident)**	
1 2 3 4 5 No answer	7 (5.6%) 6 (4.8%) 9 (7.1%) 36 (28.6%) 67 (53.1%) 1 (0.8%)

Expressed in number of answers (percentages).

## Discussion

The questionnaire for this study was developed at the beginning of 2021 and was sent to dental practitioners working in the Cantons of Vaud and Geneva situated in the French-speaking part of Switzerland. The questionnaire was sent twice between March and June 2021 in order to obtain a sufficient number of responders. The questions focused on the year 2020, which was marked by 3 periods: before the pandemic, the first and the second wave.

314 questionnaires were sent and 126 dentists responded, which corresponds to a participation rate of 40%. Although this rate seems weak, we should keep in mind that dentists were very much sollicited at that time and were under remarkable psychological stress. Other surveys have reported similar participation rates.^3,5^

When the lockdown was implemented, 77% of the participants provided emergency care, as requested by the State but also as a personal decision. Similarly an online survey involving 1731 participants from Austria, Germany, Switzerland and South Tyrol was conducted in August 2020 to explore dentists’ working conditions during the first COVID-19 pandemic lockdown.^22^ Although the participation of Swiss dentists was very low (103 participants corresponds to 6% of the total sample), it was reported that 53.7% of Germans, 45.5% of Austrians and 11.7% of Swiss reduced their opening hours, and 42.8% of Austrians, 41.5% of Swiss and 17.3% of Germans temporarily closed their facilities. Due to the potentially high risk of infection in dental settings, non-urgent dental treatment was largely suspended in the four countries.

88.1% of the partricipants did not consider themselves at risk of infection; at the time the questionnaire was sent, 83.3% of the dentists had not been infected by COVID-19. A similar survey conducted in the US during the same time period reported that the COVID-19 prevalence and the positive-testing rates were low among the practicioners (82.2% were asymptomatic).^10^ This indicates that the recommendations for enhanced infection control practices were sufficient to limit infection in dental settings. For the Swiss dentists, the guidelines were satisfactory and enabled them to implement the new treatment protocols in their practice; this was not the case in all countries, as significant knowledge gaps were recorded in similar surveys.^9,17,20^ Uncertainty about infection control measures and appropriate personal protective equipment (PPE) use, due to the lack of precise operating guidelines, was also reported by Italian dentists, at least during the first wave of the pandemic.^8^ Another large survey conducted in March 2020 including dentists from 30 different countries, reported that, although the majority of the dentists (90%) were aware of the precautions and measures necessary to continue providing dental treatment to their patients, their implementation was reported by only 61% of the participants.^1^

The main proposed measures in terms of PPE and protection of the patients were respected by the majority of dentists. Similar findings were also reported in other surveys.^10,23^ In Spain, only 25% of the dentists were equipped with a FFP2 mask, and thus were obliged to reduce and even cease their practice.^13^ In fact, those authors concluded that the Spanish geographical regions with the highest rates of COVID-19 had the least amount of individual protective resources (FFP2 and FFP3 masks).

The psychological impact of the COVID-19 pandemic on dental personnel was considerable, especially during the first wave. The major reasons for a large proportion of participants were not only finances and fear of infection, but also lack of knowledge and guidelines. During the second wave, the impact was less severe, as dentists were better informed and had received satisfactory guidelines. A similar study conducted in Norway reported a substantial psychological burden among dental personnel in terms of fear of being infected (71.9%) or of infecting others (85.4%). However, adequate infection control measures were associated with less fear of infection and feeling of insecurity.^21^

The COVID-19 pandemic has profoundly affected and is still affecting the world’s population, physically, psychologically and economically. Some sectors are more affected than others, especially health care, which is on the front line and whose personnel are at a greater risk of infection.

The transmission of the virus occurs through close contact as well as droplets, and is facilitated by the generation of aerosols, which places dental professionals at high risk of infection. This has led to great anxiety among practitioners. However, the rate of transmission in the majority of studies was not higher than in any of the other health-care sectors, which may indicate good infection control in the dental setting with appropriate guidelines.

The dental-care system was still active during the first peak of the pandemic, as 77.8% of the dentists continued to provide care despite the lockdown.

However, the psychological impacts were unavoidable, as many studies have shown. At that time, the arrival of this new, unknown virus with no treatment or vaccine available, inevitably led to widespread fear and anxiety.^6,16^

## Conclusion

This retrospective study showed that dentists were influenced by the pandemic and underwent many changes in their everyday practice. However, they managed to continue working with low rates of transmission by postponing non-emergency treatment, as well as by implementing protective measures and identifying infected patients.

While the present study was being conducted, the first vaccine arrived at the beginning of 2021, giving hope to the majority of the population as a solution to this pandemic. Today, after several waves of COVID-19 infection involving different variations of the virus, the vaccination and the natural immunity of the entire population seem to have fulfilled this hope. Evaluating the long-term physical, psychological and economic impacts of this pandemic will still require time.
